# Humanized Mouse Model Mimicking Pathology of Human Tuberculosis for *in vivo* Evaluation of Drug Regimens

**DOI:** 10.3389/fimmu.2019.00089

**Published:** 2019-01-31

**Authors:** Frida Arrey, Delia Löwe, Stefanie Kuhlmann, Peggy Kaiser, Pedro Moura-Alves, Gopinath Krishnamoorthy, Laura Lozza, Jeroen Maertzdorf, Tatsiana Skrahina, Alena Skrahina, Martin Gengenbacher, Geraldine Nouailles, Stefan H. E. Kaufmann

**Affiliations:** ^1^Department of Immunology, Max Planck Institute for Infection Biology, Berlin, Germany; ^2^Department of Molecular Pharmacology and Cell Biology, Leibniz Forschungsinstitut für Molekulare Pharmakologie, Berlin, Germany; ^3^Republican Scientific and Practical Centre for Pulmonology and Tuberculosis, Minsk, Belarus; ^4^Public Health Research Institute, New Jersey Medical School, Rutgers, The State University of New Jersey, Newark, NJ, United States; ^5^Division of Pulmonary Inflammation, Charité-Universitätsmedizin Berlin, Berlin, Germany

**Keywords:** *Mycobacterium tuberculosis*, humanized mouse models, lung, infection, granuloma, human immune system mice, antibiotics, pathology

## Abstract

Human immune system mice are highly valuable for *in vivo* dissection of human immune responses. Although they were employed for analyzing tuberculosis (TB) disease, there is little data on the spatial organization and cellular composition of human immune cells in TB granuloma pathology in this model. We demonstrate that human immune system mice, generated by transplanted human fetal liver derived hematopoietic stem cells develop a continuum of pulmonary lesions upon *Mycobacterium tuberculosis* aerosol infection. In particular, caseous necrotic granulomas, which contribute to prolonged TB treatment time, developed, and had cellular phenotypic spatial-organization similar to TB patients. By comparing two recommended drug regimens, we confirmed observations made in clinical settings: Adding Moxifloxacin to a classical chemotherapy regimen had no beneficial effects on bacterial eradication. We consider this model instrumental for deeper understanding of human specific features of TB pathogenesis and of particular value for the pre-clinical drug development pipeline.

## Introduction

Tuberculosis (TB) is still the deadliest infectious disease, globally causing 5,000 deaths every day. In 2017, this added up to 1.6 million deaths and 10 million individuals fell ill from TB (1). TB is primarily transmitted through the aerosol route and manifests as a pulmonary disease in immunocompetent individuals with signs of dissemination in immunocompromised patients and children (2). The causative agent of TB, the intracellular pathogen *Mycobacterium tuberculosis* (Mtb), is phagocytosed by alveolar macrophages upon entry into the lungs and these macrophages congregate and act as a focus of infection together with other innate immune cells such as neutrophils (3). This focus is the first step in granuloma formation, a disease pathology which is the classical hallmark of pulmonary TB (4). The granuloma acts as a means to contain the infection (5) but also as a source of dissemination to other organs via the lymphatic system (6) and newly created blood vessels (7, 8). The granuloma also serves as a source of transmission upon cavitation and rupture into the airways (9, 10). Lung TB lesions form a spectrum comprising solid non-necrotic granulomas, TB pneumonia, necrotic granulomas, and caseous necrotic granulomas with distinct microenvironments. Most mouse models of TB do not exhibit this granuloma heterogeneity (11) and murine models that do exhibit a broad granuloma range lack specific aspects of human TB (12–14). These limitations preclude studies on human immune cell trafficking, infiltration, and cellular interactions in the granuloma microenvironment.

Immuno-deficient NOD *scid* gamma (NSG) mice have emerged as one of the best-suited strains for human stem cell transplantations (15). Human immune system (HIS) NSG mice recapitulate key immuno-pathological features of major human diseases caused by viruses such as human immunodeficiency virus (HIV) (16), hepatitis C virus (17), Epstein-Barr virus (18) and bacteria such as *Salmonella typhi* (19), and *Borrelia hermsii* (20). Here, we describe the establishment of human fetal liver derived human stem cell NSG mice for analyzing pulmonary TB. We conducted immune-phenotyping, histopathology, and immunohistochemistry to determine the extent by which the developing granulomas in these mice resemble those of TB patients. Our results reveal that the HIS-NSG mice develop TB granuloma heterogeneity as seen in TB patients. More importantly, caseous necrotic granulomas expressed an immunophenotype and spatial-organization resembling what is observed in TB patients.

In recent years, there has been a push in shortening TB drug chemotherapy, as a means to improve compliance with TB treatment, reduce relapse, and restrict the emergence of drug resistant TB (21). Moxifloxacin (M), a fluoroquinolone antibiotic, has been proposed as an additive to canonical drug regimens to shorten treatment time of drug sensitive TB from six to four months (22). Taking into consideration that caseous necrotic granulomas account for reduced efficacy of anti-TB treatment, we compared the effects of M on combination therapy with isoniazid (H), rifampicin (R), and pyrazinamide (Z) (HRZ). Both regimens initially reduced lung bacterial loads within 2 weeks. However, prolonged treatment time of 4 weeks had no further beneficial effect on bacterial elimination, instead, lung bacterial loads gradually increased. This pattern could explain that Mtb is not eradicated during therapy because of impaired penetration of drugs into the granuloma caseum (23). In conclusion, HIS-NSG mice recapitulate key features of the human immune response and the corresponding pathology upon Mtb infection. Hence, it is tempting to propose HIS-NSG mice for preclinical model assessment of anti-TB drugs in an *in vivo* setting.

## Materials and Methods

### Human Lungs Tissue

Human tissue samples were collected from materials removed during lung resection type surgery in patients who underwent elective surgery on the background of individualized (optimized) TB (MDR-TB) chemotherapy at the Republican Scientific and Practical Center for Pulmonology and Tuberculosis, Minsk, Belarus. For histopathological analysis, surgical lung cuts were sliced in small pieces and fixed in 4% paraformaldehyde overnight. According to the Helsinki Declaration 2008, ethical approval was obtained by the Ethical Committee of the Republican Research and Practical Center, appointed by Director Prof. Dr. Henadz Hurevich. Republican Research and Practical Center Dolginovski Trakt, 157, 220053, Minsk, Belarus.

### Mice

All animal studies have been ethically reviewed and approved by the State Office for Health and Social Services, Berlin, Germany. Experimental procedures were carried out in accordance with the European directive 2010/63/EU on Care, Welfare and Treatment of Animals. NOD.Cg-*Prkdc*^*scid*^ *Il2rg*^*tm*1*Wjl*^/SzJ (NSG) and C57BL/6 (BL/6) mice were obtained from The Jackson Laboratory and kept and bred under specific pathogen-free conditions. Infected mice were housed in a biosafety level 3 facility under specific pathogen–free conditions.

### Generation of HIS-NSG Mice

HIS-NSG mice (male and female) were generated using the human stem cell neonate NSG protocol as previously described (18, 19). In brief, human fetal liver was obtained from Advanced Bioscience Resources, California, USA. The tissue was mechanically cut into small pieces with surgical scissors and treated with 2 mg/mL collagenase D (Roche) in Hank's balanced salt solution with CaCl_2_/MgCl_2_ (Gibco) for 30 min in a 5% CO_2_ at 37°C followed by filtering through 70-μm nylon cell strainers (BD Biosciences). CD34^+^ human hematopoietic stem and progenitor cells (HSCs) were isolated using the direct CD34 MicroBead kit (Miltenyi Biotec). One- to three day-old NSG mice were irradiated with 100 cGy and injected intrahepatically with 1–2 × 10^5^ CD34^+^ HPCs 24 h after irradiation. Some NSG mice received 50 μl of PBS as a control. The mice were bled 10–12 weeks after engraftment and peripheral lymphocytes were stained with monoclonal antibodies purchased from BioLegend: anti-mouse CD45 (clone 30-F11), anti-human CD45 (clone HI30), anti-human CD3 (clone UCHT1), anti-human CD4 (clone OKT4), anti-human CD8 (clone SK-1), and anti-human CD19 (clone HIB19) for 30 min at 4°C. After red blood cell lysis, the samples were analyzed by flow cytometry using a BD LSR II to assess reconstitution of the human immune system and visualized using FlowJo software.

### Bacteria and Infection

Mtb strain H37Rv (ATCC#27294) was grown in Middlebrook 7H9 broth (BD Biosciences) supplemented with 0.2% glycerol, 0.05% Tween 80, and 10% ADC enrichment (BD Biosciences). Mid logarithmic cultures were harvested and stored at −80°C. Stocks were tested for virulence and titrated prior to use. Twelve-week-old animals were aerosol infected with Mtb, using a Glas-Col inhalation exposure system at a low dose of approximately 15–30 CFUs per mouse. At designated time points, serial dilutions of tissue homogenates were plated onto Middlebrook 7H11 agar supplemented with 10% OADC Enrichment (BD Biosciences) and ampicillin (25 μg/ml). CFUs were determined after 3 to 4 weeks of culture at 37°C.

### Histology and Immunohistochemistry

Mice were sacrificed at specific time points and tissues for histopathologic analyses were fixed in 4% (wt/vol) paraformaldehyde at 4°C for 24 h. Human and mouse tissues were processed as follows. Two- to three-μm thick formalin-fixed, paraffin-embedded tissue sections were deparaffinized and rehydrated and subsequently stained with either hematoxylin and eosin stain or a modified Ziehl-Neelsen stain for bacterial detection or immunohistochemistry stain, respectively (24). For immunohistochemistry, tissue sections underwent a steam pressure antigen retrieval step using Dako target retrieval solution, pH9. Slides were incubated with the following mouse primary antibodies: anti-human CD68 (clone KP1, Dako), anti-human CD15 (BD), anti-human CD4 (clone 4B12, Leica Biosystems), anti-human CD8 (clone 4B11, Leica Biosystems), and anti-human CD20 (clone L26, Leica Biosystems) for 1 h at room temperature in a humidified chamber. The tissue sections were developed with a biotin-free HRP- polymer system (MACH 4 Universal HRP-polymer kit, Biocare Medical) and DAB substrate (Dako). Tissue sections were imaged using a Leica DRMB microscope with a ProgResC12 (Jenoptik) Camera. Lung tissue sections were scanned using Aperio AT2 Leica slide scanner. Further analysis was carried out using ImageJ version 1.41.

### Cell Isolation From Tissues

A portion of the perfused right lung lobes was mechanically digested and incubated for 30 min at 37°C at 5% CO_2_ in RPMI 1640 medium (Gibco) supplemented with glutamine, Na-pyruvate, 2-ME, penicillin, streptomycin, 10% heat-inactivated FCS, collagenase D (Roche), and collagenase type VIII (Sigma-Aldrich). Single-cell suspensions of lungs were then prepared by meshing the lungs through 40-μm nylon cell strainers and red blood cell lysis. Viable cells were counted by trypan blue exclusion. Cells were blocked with anti-mouse CD16/CD32 mAb (BioLegend) and human Fc receptor blocking solution (BioLegend), and then surface stained with monoclonal antibodies purchased from BioLegend: anti-mouse CD45 (clone 30-F11) anti-human CD45 (clone HI30), anti-human CD3 (clone UCHT1), anti-human CD4 (clone OKT4), anti-human CD8 (clone SK-1), anti-human CD19 (clone HIB19), anti-human CD20 (clone 2H7), anti-human CD33 (clone WM53), anti-human CD66a/c/e/b (clone ASL-32 and G10F5), anti-human CD14 (clone M5E2), and anti-human CD11c (clone Bu15) for 30 min at 4°C. The samples were analyzed by flow cytometry using a BD FACS CANTO II in the biosafety level 3 and visualized using FlowJo software.

### Human Biomolecule Characterization in Lung Tissues of HIS-NSG Mice

A portion of the perfused right lung lobes was homogenized in 700 μl of lysis buffer with protease inhibitor (Roche) at specific time points. Homogenized tissue was centrifuged at 6,708 × g and the supernatants were spun through 0.22 μm Spin-X filter tubes and stored in −80°C. Human cytokines, chemokines, growth factors, and antibody concentrations were analyzed in the supernatants by using Bio-Plex Pro Human Cytokine 27-plex Panel and Bio-Plex Pro Human Isotyping Panel (Bio-Rad). The data were acquired and analyzed on a Bio-Rad Bio-Plex 200 system.

### Drug Treatment and Bacterial Load Enumeration

Cord blood-engrafted HIS-NSG mice were purchased from Jackson Laboratory. Briefly, Jackson laboratory injected human CD34^+^ stem cells i.v. into myeloablated NSG mice and 12 weeks later humanization was confirmed by presence of >25% huCD45+ in the peripheral blood. These HIS-NSG mice were Mtb infected at 12 weeks of age. Isoniazid (H: 30 mg/kg), rifampicin (R: 10 mg/kg) and pyrazinamide (Z: 125 mg/kg) (HRZ), and moxifloxacin (M: 100 mg/kg) were purchased from Sigma or ChemPacific. The regimens were formulated in 200 μl 0.4% methyl cellulose and administered 6 days per week by oral gavage starting at day 28 p.i. At 2 and 4 weeks after treatment start, serial dilutions of tissue homogenates were plated onto Middlebrook 7H11 agar supplemented with 10% OADC Enrichment (BD Biosciences). To reduce the effect of drug-carry over 0.4% w/v activated charcoal was added to Middlebrook 7H11 agar. CFUs were determined after 3–4 weeks of culture at 37°C.

### Statistical Analysis

PRISM GraphPad software was used for statistical analyses. Survival curves were analyzed using Kaplan-Meier method and log-rank (Mantel-Cox) test. Two groups were compared using Mann-Whitney *U* test for non-parametric data, more than two groups using Kruskal-Wallis/Dunn's multiple comparisons test (non-parametric) and grouped analyses were performed using 2-way ANOVA/Dunnett's multiple comparison test. *P*-values smaller than 0.05 were considered significant.

## Results

### NOD Scid Gamma Mice Are Efficiently Human Immune Reconstituted by Human Stem Cells

To generate a HIS mouse model for TB, we implemented a NSG mouse breeding program where the resulting newborn (1–3 days old) pups received human fetal liver CD34^+^ HSCs via intra-hepatic injection after 100 cGy sublethal irradiation. Depending on size and age of the fetal liver (16–24 weeks), cohorts of 10–50 HIS-NSG mice were generated per individual donor graft. Simultaneously, some of the NSG littermates underwent the same conditioning but received PBS (PBS-NSG). The mice were bled 10 weeks post engraftment and peripheral leukocytes were analyzed to assess human immune cell reconstitution (Supplementary Figure 1A). In HIS-NSG mice, we observed average frequencies of 55% human CD45^+^ leukocytes in the blood by 3 months (Supplementary Figure 1B), although with variability between each mouse and donor graft. These cells included granulocytes, monocytes and lymphocytes, comprising CD4^+^ helper T cells, CD8^+^ cytotoxic T cells, and CD19^+^ B cells (Supplementary Figure 1B) in line with previous findings (18, 25). To determine the extent of human immune cell reconstitution and penetrance in the lungs, HIS-NSG mice were sacrificed and lung tissue sections were immunostained using human specific antibodies. Naïve HIS-NSG lung tissue morphology showed a sponge like appearance with large alveolar spaces (Supplementary Figure 1C) and a spatial distribution of alveolar and parenchymal CD68^+^ macrophages (Supplementary Figure 1D). CD15^+^ neutrophils, CD3^+^ T cells, and CD20^+^ B cells were located in the lung parenchyma (Supplementary Figure 1D). Altogether, these data demonstrate that reconstitution of the human immune system endows HIS-NSG mice with human immune cells playing key roles in TB pathology.

### Mtb Infected HIS-NSG Mice Display Features of TB Pathology

**Figure 1:**
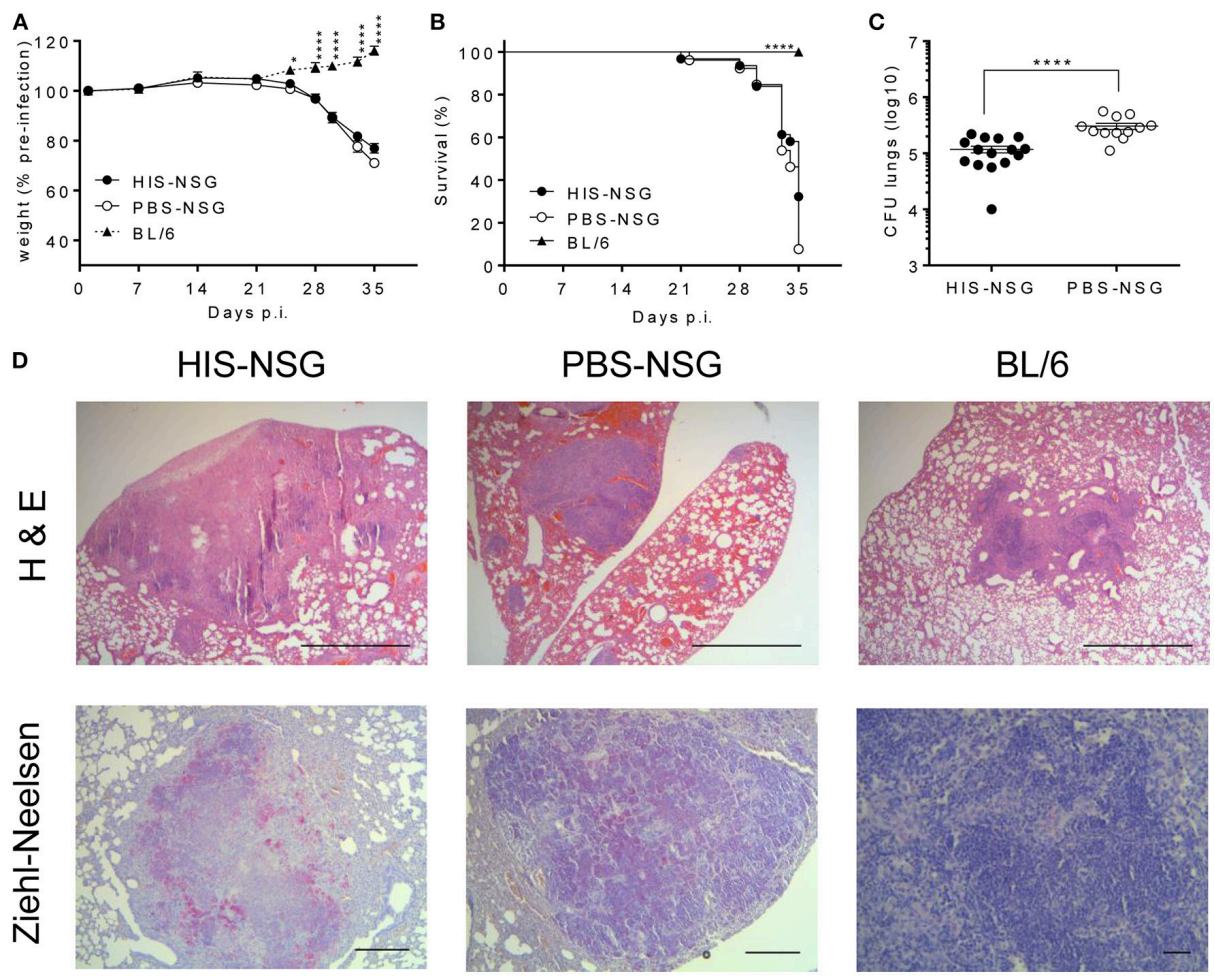
HIS-NSG mice are susceptible to aerosolized Mtb and develop lung lesions upon Mtb infection. HIS-NSG mice, PBS-NSG mice and BL/6 mice were infected with low dose aerosol infection with Mtb (~20 CFUs). **(A)** Relative weight curves, 2-way ANOVA/Dunnett's multiple comparisons test, comparison to HIS-NSG mice, mean ± SEM. **(B)** Kaplan-Meier curves showing survival, log-rank test. **(C)** Bacterial burden in lungs of mice at day 35 p.i. Mann-Whitney *U* test, mean ± SEM. **(A–C)** Data were pooled from three independent experiments. ^****^P < 0.0001. **(D)** Microscopic analysis (Hematoxylin and Eosin; 25X, scale bar = 1,000 μm) of lesions in representative HIS-NSG, PBS-NSG, and BL/6 lungs were performed in addition to mycobacterial identification (Ziehl-Neelsen; 50X, scale bar = 500 μm, BL/6 lung at 200X, scale bar = 100 μm) at day 35 post Mtb infection. Data are representative of three independent experiments.

To determine whether the HIS-NSG mice develop general characteristics of TB, we infected them with low dose aerosolized Mtb H37Rv (15–30 CFU) together with PBS-NSG and C57BL/6 (BL/6) mice as controls. HIS-NSG and PBS-NSG mice exhibited clinical TB symptoms such as lethargy and weight loss, starting from day 25 post infection (p.i.) (Figure 1A). In contrast to NSG groups and as previously reported, BL/6 mice maintained and even gained weight from day 25 p.i. (24, 26). Despite successful engraftment, HIS-NSG mice succumbed to infection by day 33 to day 35 p.i., comparable to PBS-NSG mice (Figure 1B). Disease susceptibility of HIS-NSG mice, due to their genetic immune-deficient background, fetal stem cell origin and inexperienced immune system likely resemble more to pediatric and immunocompromised individuals than healthy adults, indeed, inferior survival and TB outcome has been described for these individuals (27). Despite low dose aerosolized Mtb infection, HIS-NSG, and PBS-NSG mice displayed high bacterial numbers in lungs (Figure 1C) as well as mycobacterial dissemination into other organs including spleen, liver, kidney, and bone marrow (Supplementary Figure 2A). Notably, HIS-NSG mice harbored high, but significantly lower bacterial loads in the lungs, as compared to PBS-NSG mice (Figure 1C), suggesting that the human immune cells in HIS-NSG mice participated in pulmonary immune defense against Mtb.

**Figure 2:**
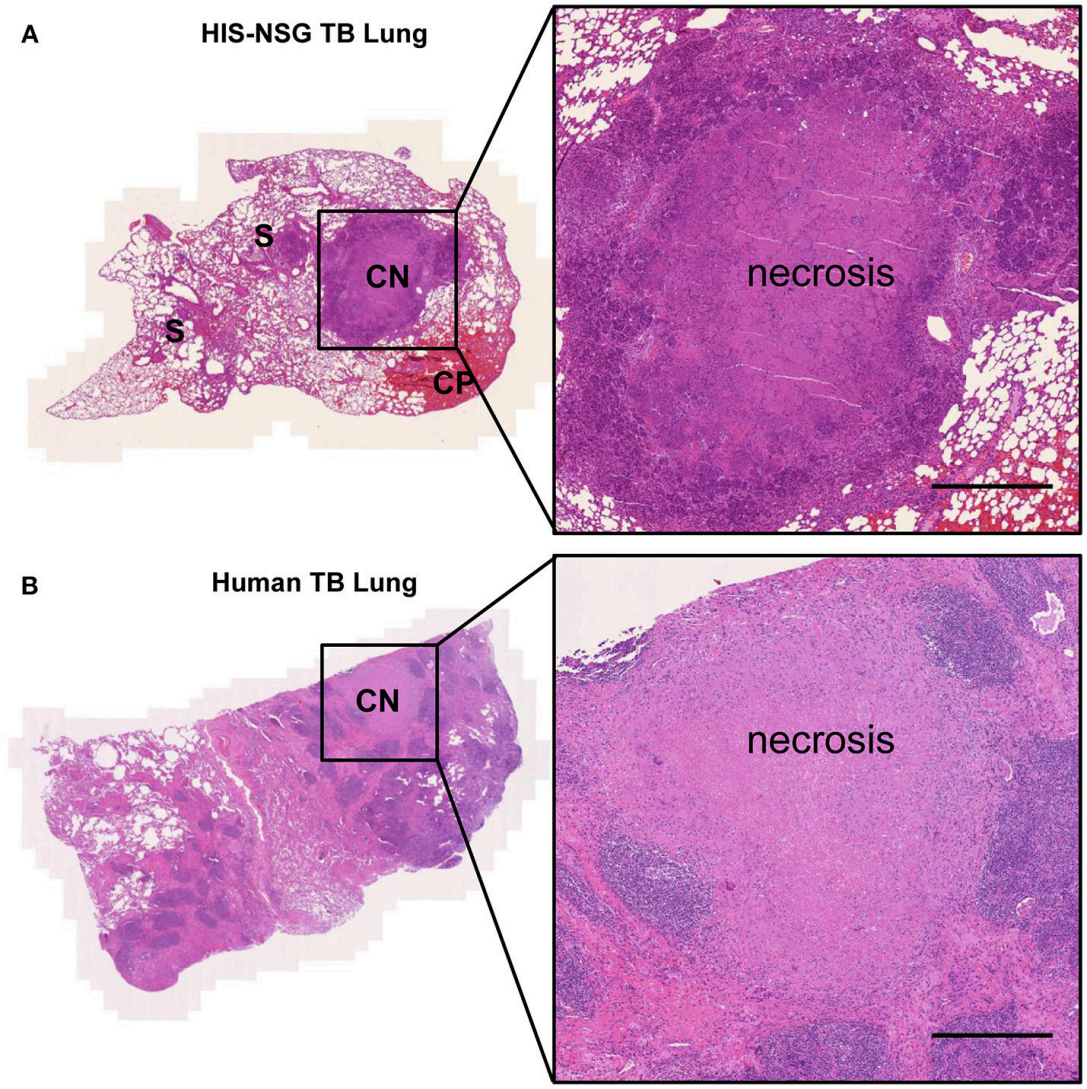
HIS-NSG mice develop human like caseous necrotic granulomas. **(A)** Lesions in representative Mtb infected HIS-NSG lung sections at day 35 p.i., S, solid non-necrotic granulomas; CP, coalesced parenchyma; CN, caseous necrotic granuloma. **(B)** Representative tissue cuts from a TB patient's lungs. Tissue slide scan, Hematoxylin and Eosin 50X, scale bar = 500 μm.

Next, we interrogated whether disease pathology in HIS-NSG mice following Mtb infection was similar to human TB. To this end, we performed *post-mortem* macroscopic and microscopic examination of organs around day 35 p.i. (animals reached experimental human endpoint criteria and were euthanized). All three groups had lung lesions evident at macroscopic examination (Supplementary Figure 2B). Microscopic examination of H&E staining, however, revealed differences: lung lesions in BL/6 and PBS-NSG mice comprised of non-necrotic cellular aggregates (Figure 1D). In contrast, HIS-NSG lungs contained caseous necrotic granulomas with a core consisting of necrotic debris and an outer cellular cuff (Figures 1D, 2A). Ziehl-Neelsen staining identified Mtb in non-necrotic aggregates in BL/6 and PBS-NSG mice with fewer numbers in BL/6 compared to PBS-NSG mice. Caseous necrotic granulomas of HIS-NSG mice displayed extracellular bacteria in the necrotic core and apparent intracellular ones in the cuff (Figure 1D). Furthermore, in addition to caseous necrotic granulomas, HIS-NSG lungs presented small non-necrotic cellular aggregates and coalesced lung parenchyma (Figure 2A). Importantly, the pathology of caseous necrotic granulomas resembled what is being observed in active TB patients (Figures 2A,B).

Visual examination revealed white spots in Mtb-infected HIS-NSG and PBS-NSG spleens (Supplementary Figure 3A), while BL/6 spleens were unspotted. Microscopic examination unveiled centers of splenic caseous necrotic granulomas full of extracellular bacteria in HIS-NSG mice, whereas in PBS-NSG mice non-necrotic splenic aggregates consisted of innate leukocytes with bacteria (Supplementary Figures 3B,C). There were no splenic granulomas in the BL/6 mice, with a few bacteria interspersed throughout the spleen (Supplementary Figures 3B,C). Collectively, upon Mtb infection HIS-NSG mice developed granulomas resembling human-like pathology with dissemination most similar to pediatric and immunocompromised TB.

### Caseous Necrotic Granulomas in HIS-NSG Mice Display Neutrophil Rich Centers

**Figure 3:**
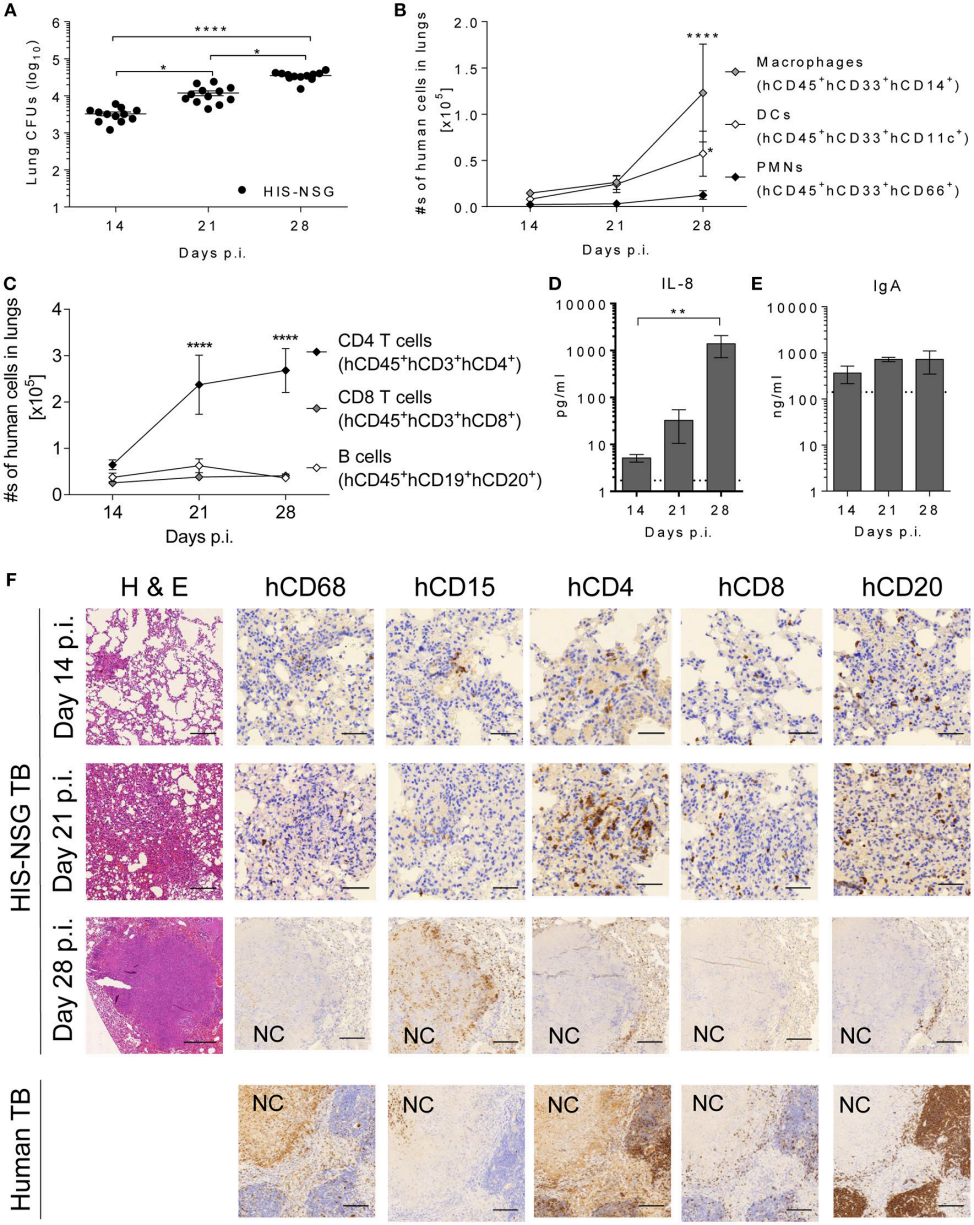
Human immune cells are recruited and develop a human-like granuloma organization after Mtb infection. **(A)** Bacterial burden was determined at days 14, 21, and 28 p.i. in HIS-NSG lungs. Data were pooled from two independent experiments, *n*_pooled_ = 12 per time point, Kruskal Wallis/Dunn's multiple comparisons test. Line graphs showing the absolute numbers of **(B)** human innate immune cells and **(C)** human adaptive immune cells infiltrating HIS-NSG lungs at 14, 21, and 28 days p.i. Data pooled from one to three experiments, *n* = 6–12 mice per time point, 2-way ANOVA/Dunnett's Multiple comparisons test, effect within population compared to day 14 p.i. Bar graphs showing **(D)** IL-8, and **(E)** human IgA protein levels at 14, 21, and 28 days p.i. Dotted line: detection limit. *n* = 4–5 mice per time point, Kruskal-Wallis/Dunn's multiple comparisons test. **(A–E)** Mean ± SEM, ^****^P < 0.0001, ^**^P < 0.01, ^*^P < 0.05. **(F)** Microscopic analysis (Hematoxylin and Eosin 100X, scale bar = 200 μm) in representative HIS-NSG lungs at 14, 21, and 28 days p.i. was carried out. In addition, human immune-stains of macrophages (CD68), neutrophils (CD15), CD4^+^ T cells (CD4), CD8^+^ T cells (CD8), and B cells (CD20) were also performed in Mtb-infected HIS-NSG lungs and human TB lungs. NC, necrotic core (days 14 and 21 p.i., 400X, scale bar = 50 μm, day 28 p.i. and human TB lung, 100X, scale bar = 200 μm). Data are representative of three independent experiments, *n* = 3.

To characterize local responses in the humanized lungs during Mtb infection, we assessed the spatial organization of human immune cells, expression of different biomolecules, and bacterial burden in HIS-NSG mice at 14, 21, and 28 days p.i. On day 14 p.i., infection was well-established in the lungs and bacterial loads further increased 4 fold by day 28 p.i. (Figure 3A). As measured by flow cytometry (Supplementary Figure 4), total numbers of human neutrophils (CD45^+^CD33^+^CD66^+^), human DCs (CD45^+^CD33^+^CD11c^+^), and human monocytes/macrophages (CD45^+^CD33^+^CD14^+^) in the lungs markedly increased over this time frame (Figure 3B). Likewise, increasing numbers of pulmonary human helper T cells (CD45^+^CD3^+^CD4^+^), human cytotoxic T cells (CD45^+^CD3^+^CD8^+^), and human B cells (CD45^+^CD19^+^CD20^+^) were detected (Figure 3C). Differences in the expression of immune/inflammatory biomolecules known to be involved in the recruitment and activation of immune cells crucial in human granuloma formation and development, were also observed. Expression of pro-inflammatory mediators (IL-1β, IL-6, MCP-1, MIP-1β, IP-10) in lung homogenates increased from day 14 to day 28 p.i. (Supplementary Figure 5). Notably, human-specific neutrophil chemoattractant IL-8 increased as TB disease progressed (Figure 3D). Finally, levels of human mucosal IgA antibodies were elevated in infected HIS-NSG lungs (Figure 3E). The predominantly pro-inflammatory response observed was consistent with previous studies in other animal models of TB and in lung samples from human TB patients (28). Hence, our results reveal a basic functional immune response to Mtb in the HIS-NSG lungs.

Biomolecules influence cellular spatial organization of leukocytes (28). To confirm human-like TB pathology and granuloma organization on a cellular level, lung sections were stained for innate and adaptive immune cells. At day 14 p.i. most HIS-NSG lungs contained small non-necrotic myeloid aggregates, surrounded by lymphocytes and normal healthy tissue (Supplementary Figure 6). At day 21 p.i., coalesced parenchyma known as TB pneumonia had developed, while at day 28 p.i., lesions consisted of large non-necrotizing and caseous necrotic granulomas with smaller adjacent caseous necrotic granulomas (Supplementary Figure 6). We conclude that the HIS-NSG mouse has the ability to model human-like lung granuloma initiation and formation. Sequential lung tissue sections were also stained for human leukocytes, including CD68^+^ macrophages, CD15^+^ neutrophils, CD4^+^ T cells, CD8^+^ T cells, and CD20^+^ B cells to visually validate and localize the cellular infiltration observed by flow cytometry (Figure 3F). As pathology progressed, the lung infiltrates became increasingly organized with macrophages and neutrophils in the center, more macrophages and neutrophils generating an inner rim followed by T cells and B cells forming an outer cuff by day 28 p.i. In particular, at day 28 post Mtb infection, caseous necrotic granulomas in the HIS-NSG mice harbored cell populations that were organized in a pattern similar to that observed in patients with active pulmonary TB (Figure 3F). We conclude that the HIS-NSG mouse mimics human TB lung granuloma formation and development, intertwined with the associated human pulmonary immune response.

### Mtb Infected HIS-NSG Mice for Drug Testing: Moxifloxacin Confers No Added Advantage

**Figure 4:**
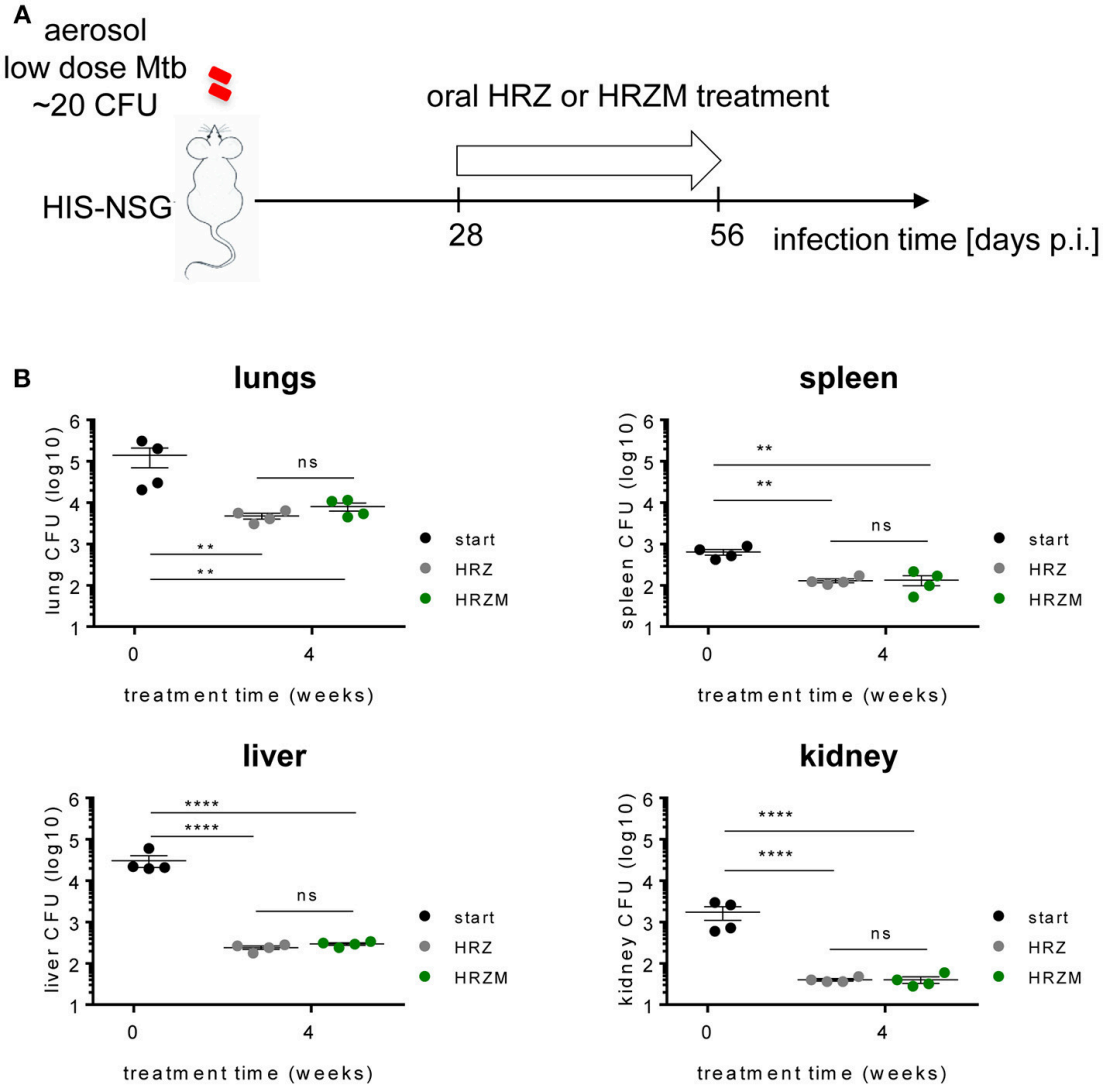
Moxifloxacin confers no added advantage for treatment of Mtb infected HIS-NSG mice. **(A)** Treatment scheme of Mtb infected HIS-NSG mice. Treatment regimen of either HRZ or HRZM combinations was initiated at day 28 p.i. (treatment start) for a period of 4 weeks. **(B)** Bacterial burden in lungs, spleen, liver and kidney, *n* = 4, means ± SEM, one way ANOVA/Tukeys's multiple comparisons test on log transformed data; ns, non significant, ^**^P < 0.01, ^****^P < 0.0001.

We applied the humanized mouse model for drug testing. We used commercially available cord-blood engrafted HIS-NSG mice from Jackson Laboratory USA, which have similarities in their humanized immune response to human fetal liver engrafted mice (29, 30). We administered two different drug combinations to Mtb infected HIS-NSG mice from day 25 p.i., as depicted in Figure 4A, to assess the utility of the HIS-NSG mice for preclinical drug studies. The classical drug regimen HRZ comprising H, R, and Z was compared to the M supplemented experimental regimen HRZM. The drugs were administered 6 days per week by oral gavage. The total number of bacilli residing in lungs of HIS-NSG mice was ~10^5^ CFU at treatment start (day 25 p.i.). After 2 weeks of treatment and independent of the regimen applied, the bacterial burden initially declined by 1–2 log (~10^3^ CFU). However, after a total of 4 weeks treatment time bacterial burden re-increased to ~10^4^ CFU at both regimens (Figure 4B). There was no significant difference between the two treatment modalities at either time point. We also assessed treatment efficacy on bacterial burden in spleen, liver and kidney, as these organs are sites of infection in pediatric and immunocompromised TB patients. Mirroring the lungs, bacterial burdens decreased and then slightly increased during the treatment course in these organs (Figure 4B).

## Discussion

Lung TB granuloma pathology is heterogeneous, not only amongst individuals, but also in each individual patient (11). Granuloma heterogeneity has been observed in Mtb infected non-human primates (NHPs) (31), guinea pigs (32), rabbits (33), Nos2^−/−^ mice (24), and C3HeB/FeJ mice (34), however it is virtually missing in traditional inbred mouse strains. Additionally, several unique pathologic features of TB (35, 36) and HIV-TB comorbidity (37) in humans cannot be examined in these models. On the other hand, small rodents are of relatively low cost, immunologically well-characterized, and a remarkable analytical toolbox exists. To this end, we have established a murine model containing human immune cells that does develop human-like caseous necrotic granulomas as TB disease progresses. TB granulomas in these HIS-NSG mice comprised solid non-necrotic granulomas, tuberculoid pneumonia, and caseous necrotic granulomas. Furthermore, heterogeneous granuloma pathology in lungs of individual HIS-NSG mice and across various stem cell donors was observed. These human-like granulomas displayed an immunophenotype and spatial organization reminiscent of pathological samples from human TB patients and they were most similar to granulomas from pediatric or immunocompromised TB patients (35).

Upon infection, HIS-NSG mice had significantly lower lung bacterial burdens in comparison to PBS-NSG mice, suggesting that the human immune system partially controlled bacterial replication. HIS-NSG mice lack HLA molecules for antigen presentation (38) and certain human cytokines (39). Thus, the human antigen presenting cells generated in this system may function sub-optimally in presenting Mtb antigens to human T cells, thereby compromising control of Mtb by adaptive immunity. As a corollary, despite successful engraftment of human immune cells in HIS-NSG mice, these animals succumbed to active TB disease around the same time post-Mtb infection as PBS-NSG mice. This period of 35 days did not allow for the formation of fibrotic granulomas (5, 35) which have been associated with latent TB infection.

The number of neutrophils in the lungs likely contributed to increased susceptibility to Mtb in HIS-NSG mice (40). To our knowledge, our HIS-NSG mouse model is the first to reveal increased levels of human-specific neutrophil attractant IL-8, which were correlated with recruitment of human neutrophils and were apparently associated with destructive tissue pathology during the course of infection. The presence of murine neutrophils of Mtb-infected HIS-NSG and PBS-NSG lungs is likely. Our earlier studies have shown that Mtb induces the secretion of neutrophil-chemoattractant CXCL5 by lung epithelial cells (26). Pulmonary epithelial cells remain of murine origin in the HIS-NSG mouse and likely remain functionally active. The murine neutrophils present in NSG mice, could thus follow the CXCL5 chemokine gradient into the lungs. Murine CXCR2 ligands, like CXCL5, can induce migration of human neutrophils *in vitro*. However, the increase in pulmonary human neutrophils strongly correlated with the presence of pulmonary human IL-8, arguing against a profound chemokine crosstalk between murine and human cells *in vivo*. Moreover, in various TB mouse studies (26, 41, 42) we showed that murine neutrophils are detrimental and cause lung pathology. Murine neutrophils could thus contribute to the observed susceptibility of HIS-NSG and PBS-NSG to low dose Mtb infection. Retrospectively, we would therefore recommend including markers for murine neutrophils in the analysis and possibly consider murine neutrophil depletion as a means to increase resistance of HIS-NSG mice in TB. A recent study with Mtb infected cynomolgus macaques (43) showed that granzyme-B expressing neutrophils correlated with higher bacterial loads in lung granulomas suggesting that they promote Mtb replication. This feeds into the previously described dualistic role of neutrophils in TB (44). Thus, neutrophils and notably myeloid derived neutrophilic suppressor cells (45) that have been shown to exacerbate TB pathogenesis should be considered as targets for host directed therapy (HDT) in TB.

In parallel with elevated B and T cell numbers in the lymphocyte cuff, IgA, and IgM were detected in the lung microenvironment. In humans, the role of B cells and antibodies in TB remains controversial (46–48), and antibodies are likely protective or detrimental depending on the disease state. Even though significant numbers of human CD4^+^ and CD8^+^ T cells accumulated in the HIS-NSG Mtb infected lungs, the bacterial burden was high. This could be due to the impaired antigen specific responses by the granuloma associated CD4^+^ (49) or CD8^+^ T cells (50) and their putative failure to induce appreciable amounts of the TB-protective cytokine IFN-γ (51). Likewise, the lack of human HLAs and poor human and murine cytokine cytokine-receptor crosstalk, particularly of IL-12 and IFN-γ (52) contributed to poor adaptive immune activation. Moreover, the balance of pro- and anti-inflammatory cytokines in the HIS-NSG lung was skewed toward the Th1 like pole with a more pro-inflammatory signature linked to higher bacterial burden (28). Future studies with NSG strains that have endowed features (15) such as transgenic human cytokines and human HLA molecules will allow to more precisely assess the Mtb specific responses of the various human immune cells.

A caveat of the HIS-NSG mouse model is the presence of a residual innate immune system, endothelium, epithelium, and stroma of murine origin (53). Although the generated human mononuclear phagocytic network readily engulfs the vast majority of Mtb, certain residual murine cells can also engulf bacteria (54). In addition, inadequate antigen presentation by murine cells to the human T cells can cause anergy (15). This “hybrid species” phagocytosis and antigen presentation could influence the ensuing immune response thereby affecting the pathology analyzed in this study. Certain hallmarks of TB pathology, such as cavitary granulomas and fibrosis, were not detected in our model, which is consistent with other humanized mouse studies using Mtb or other mycobacteria (54–56). The lack of cavitary granulomas could be due to the short experimental observation window of 35 days or due to the single low dose infection with Mtb H37Rv employed in this study, compared to multiple reinfections with clinical strains in a high exposure environment, such as household contact (57). Other possible underlying factors include the lack of human HLAs, thus HIS-NSG mice do not develop a fully competent human adaptive immune system. Their immune system rather resembles those of pediatric and immunocompromised individuals. Indeed cases of cavitary TB are rare in children (58). Furthermore, distinct clinical strains of Mtb can induce slightly different immune responses, which could generate different granulomas (59). Each granuloma has a unique microenvironment with certain cellular and bacterial phenotypes, and this variability affects antibiotic efficacy.

Oral administration of HRZ and HRZM drug regimens reduced lung bacterial loads at similar rates, demonstrating that the HIS-NSG model is feasible for preclinical TB drug evaluation. Our model revealed that the HRZM regimen did not perform better than the HRZ regimen, in contrast to previous *in vitro* data (60) which indicated that addition of M could improve TB treatment. A large human trial revealed no additional benefit when either ethambutol or H were replaced by M in standard regimens (22, 36). That M becomes less effective in the presence of human-like lung pathology was also shown in C3HeB/FeJ (61) and Nos2^−/−^ (62) mouse models of granuloma TB. However, HIS-NSG mice like the Nos2^−/−^ mice (62) had homogenous bacterial loads and similar forms of granuloma heterogeneity post Mtb infection in contrast to the C3HeB/FeJ mice (34, 61), giving the HIS-NSG mice an advantage of consistent reproducibility. In order for drugs to be effective in the granuloma especially the caseous necrotic granuloma, they need to traverse different cell layers. Low penetrance and diffusion rates into the caseum of M has been shown (63). The observed increase in bacterial burden under therapy from 2 to 4 weeks generally suggests replication of bacteria in the caseous necrotic core of the granuloma along with poor penetrance of the applied drugs. Thus, we posit that caseous necrotic granulomas of HIS-NSG mice mimick human TB caseous necrotic granulomas during drug treatment. Provided that similarities between experimental TB drug treatment regimens in HIS-NSG mice and human trials can be validated, this mouse model will qualify as a late pre-clinical gating point for the decision whether to progress into clinical trial.

Another advantage of the HIS-NSG mouse model for preclinical drug studies is the known spatial location of human immune cells in the granuloma. As earlier stated, granulomas comprise various types, densities and locations of immune cells throughout the granuloma microenvironment similar to tumors (64). In tumors, larger numbers of CD8^+^ cytotoxic T cells in the tumor margin and center correlated with better prognosis and provided a better target for enhancing antitumor responses by cancer immunotherapy (65). Therefore, future drug studies in HIS-NSG mice geared toward enhancing antibiotic effectiveness could incorporate HDTs, e.g., targeting CD8^+^ T cells and their associated factors such as human programmed cell death protein 1 (PD1) and cytotoxic T lymphocyte associated protein 4 (CTLA4) (66).

In summary, our findings qualify the HIS-NSG mouse as a preclinical model for human TB and HIV-TB as well as for testing novel intervention strategies.

## Data Availability Statement

The raw data supporting the conclusions of this manuscript will be made available by the authors, without undue reservation, to any qualified researcher.

## Author Contributions

FA, GN, and SK contributed conception and design of the study. FA, LL, JM, and GN designed and performed flow cytometric analysis. FA, PM-A, MG, and GK designed and performed drug study. FA, DL, SK, PK, and GN designed and performed experiments. FA and GN analyzed data. FA wrote the first draft of the manuscript. GN and SK wrote sections of the manuscript. PM-A, GK, LL, JM, MG, GN, SK, TS, and AS provided constructive input and editorial suggestions. All authors contributed to manuscript revision, read, and approved the submitted version.

### Conflict of Interest Statement

SK is currently employed by company Bayer Pharmaceuticals, Berlin, Germany. TS is currently employed by company Immunopep, Berlin, Germany. The remaining authors declare that the research was conducted in the absence of any commercial or financial relationships that could be construed as a potential conflict of interest.
