# Supplementary material for: Humanized Mouse Model Mimicking Pathology of Human Tuberculosis for in vivo Evaluation of Drug Regimens
Source: Front Immunol. 2019 Jan 31;10:89. doi: 10.3389/fimmu.2019.00089 (PMC6365439; doi:10.3389/fimmu.2019.00089)
Supplement: Supplementary file 1 [file Data_Sheet_1.docx]

Supplementary Material

Humanized mouse model mimicking pathology of human tuberculosis for *in vivo* evaluation of drug regimens

Frida Arrey, Delia Löwe, Stefanie Kuhlmann, Peggy Kaiser, Pedro Moura-Alves, Gopinath Krishnamoorthy, Laura Lozza, Jeroen Maertzdorf, Tatsiana Skrahina, Alena Skrahina, Martin Gengenbacher, Geraldine Nouailles, Stefan H.E. Kaufmann^*^

*** Correspondence:** Stefan H.E. Kaufmann: [kaufmann@mpiib-berlin.mpg.de](mailto:kaufmann@mpiib-berlin.mpg.de)


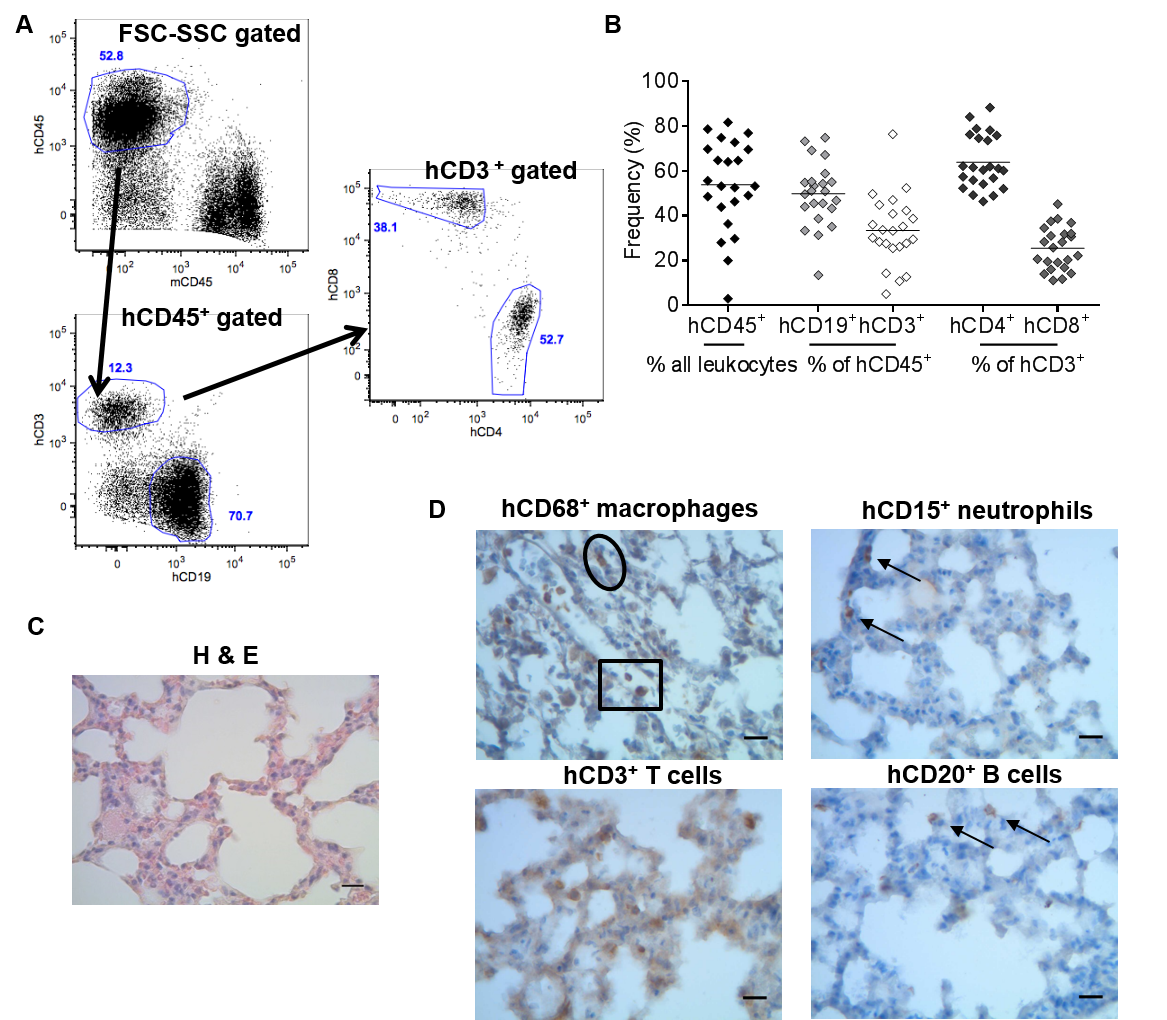


**Supplementary Figure 1. Human immune cell reconstitution in peripheral blood and lungs.** (A) Representative dot plots showing flow cytometric gating strategy applied to identify human leukocyte populations (FSC/SSC leukocytes → human CD45^+^ leukocytes → human CD3^+^ T cells/human CD19^+^ B cells → human CD4^+^ T cells and human CD8^+^ T cells). (B) Percentages of immune cells in peripheral blood of HIS-NSG mice 10 weeks post-transplantation (*n* = 23) reconstituted from the same donor are shown. Each symbol represents an individual mouse. Frequencies of CD3^+^ T cells and CD19^+^ B cells within human CD45^+^; and frequencies of CD4^+^ and CD8^+^ T cells amongst CD3^+^ cells are indicated. Horizontal bars represent means. (C, D) Representative tissue sections of HIS-NSG mice lungs 10 weeks post-transplantation. (C) Hematoxylin and Eosin (400X, scale bar = 50 µm). (D) Immunostaining with human CD68 (oval indicates parenchymal macrophages, box indicates alveolar macrophages), human CD15, human CD3 and human CD20. Black arrows indicate positive nuclear staining of CD15^+^ neutrophils and CD20^+^ B cells (400X, scale bar = 50 µm).

**
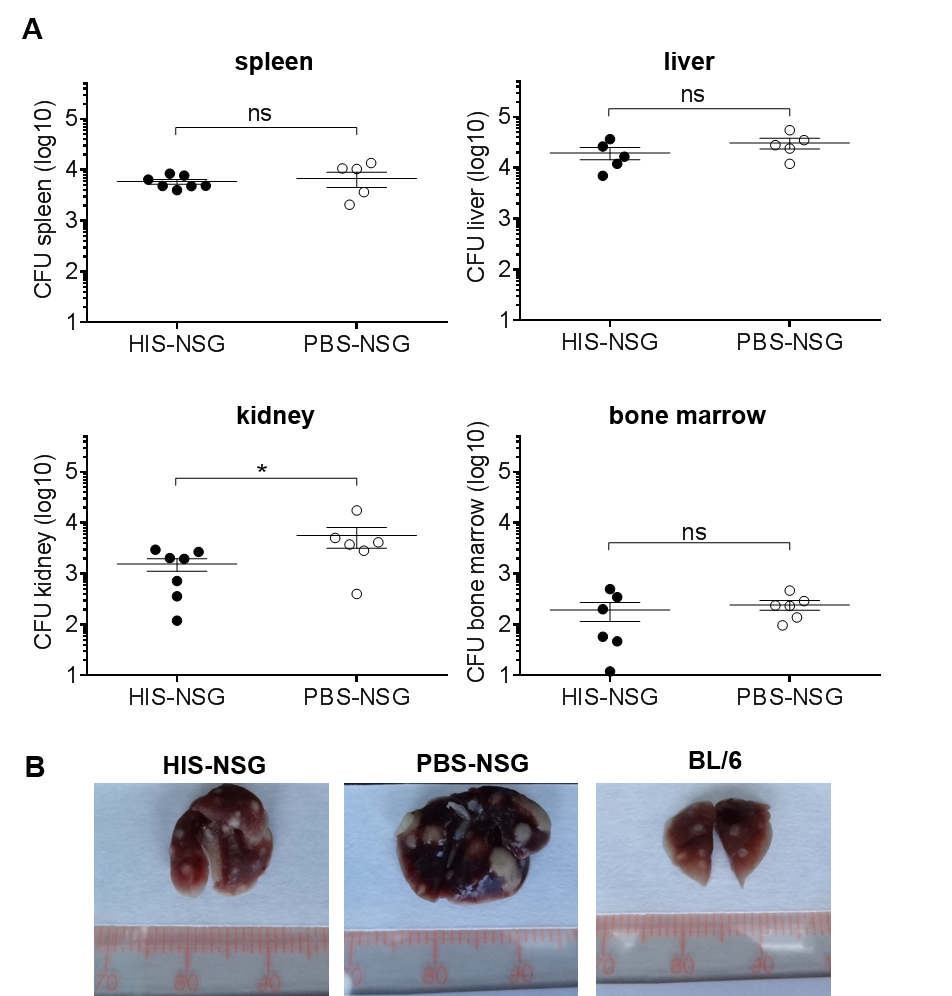
**

**Supplementary Figure 2. HIS-NSG mice are susceptible to aerosolized Mtb.** (A) Bacterial burden in spleen, liver, kidney and bone marrow at day 35 p.i. Data were pooled from 2 independent experiments, mean ± SEM, Mann-Whitney U test, ns = no significance, * *P* < 0.05. (B) Images from macroscopic (photographs) analysis of lung lesions in HIS-NSG, PBS-NSG and BL/6 lungs at day 35 p.i.. Data are representative of two independent experiments.


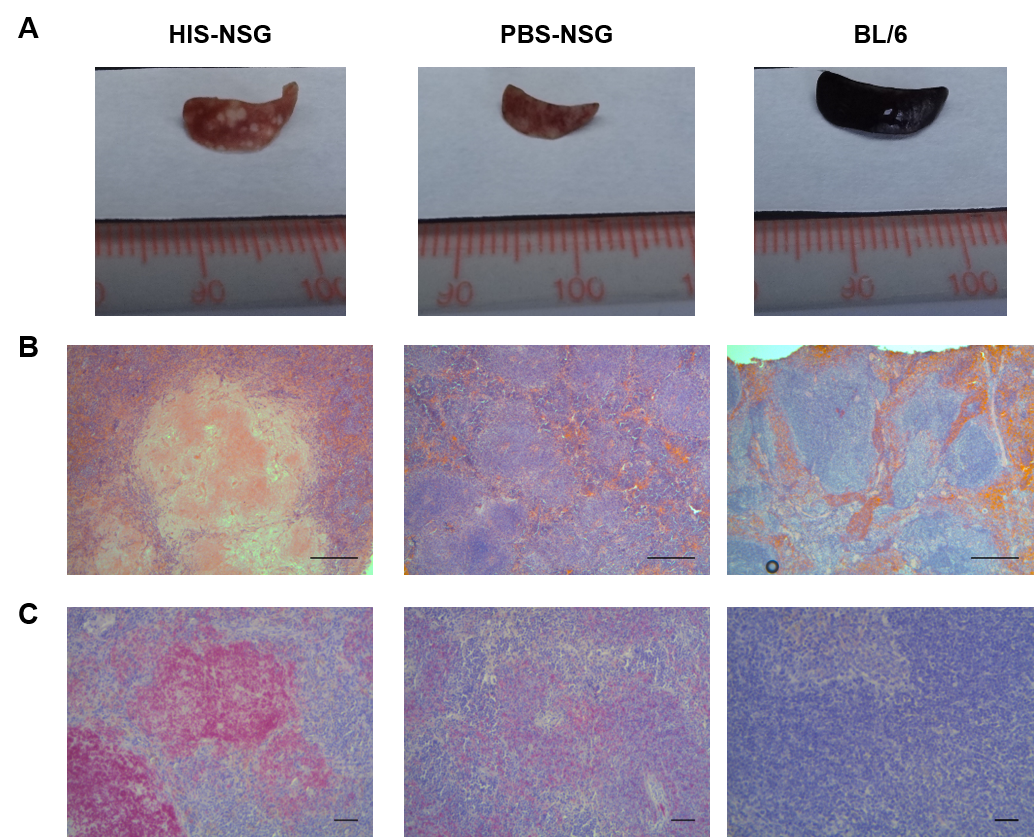


**Supplementary Figure 3. HIS-NSG mice develop splenic lesions upon Mtb infection.** (A) Macroscopic (photographs) and (B) microscopic analysis (Hematoxylin and Eosin 50X, scale bar = 500 µm) in representative HIS-NSG, PBS-NSG and BL/6 spleens at day 35 p.i. was carried out in addition to (C) mycobacterial identification (Ziehl-Neelsen 100X, scale bar = 200 µm). Data are representative of two independent experiments.

**
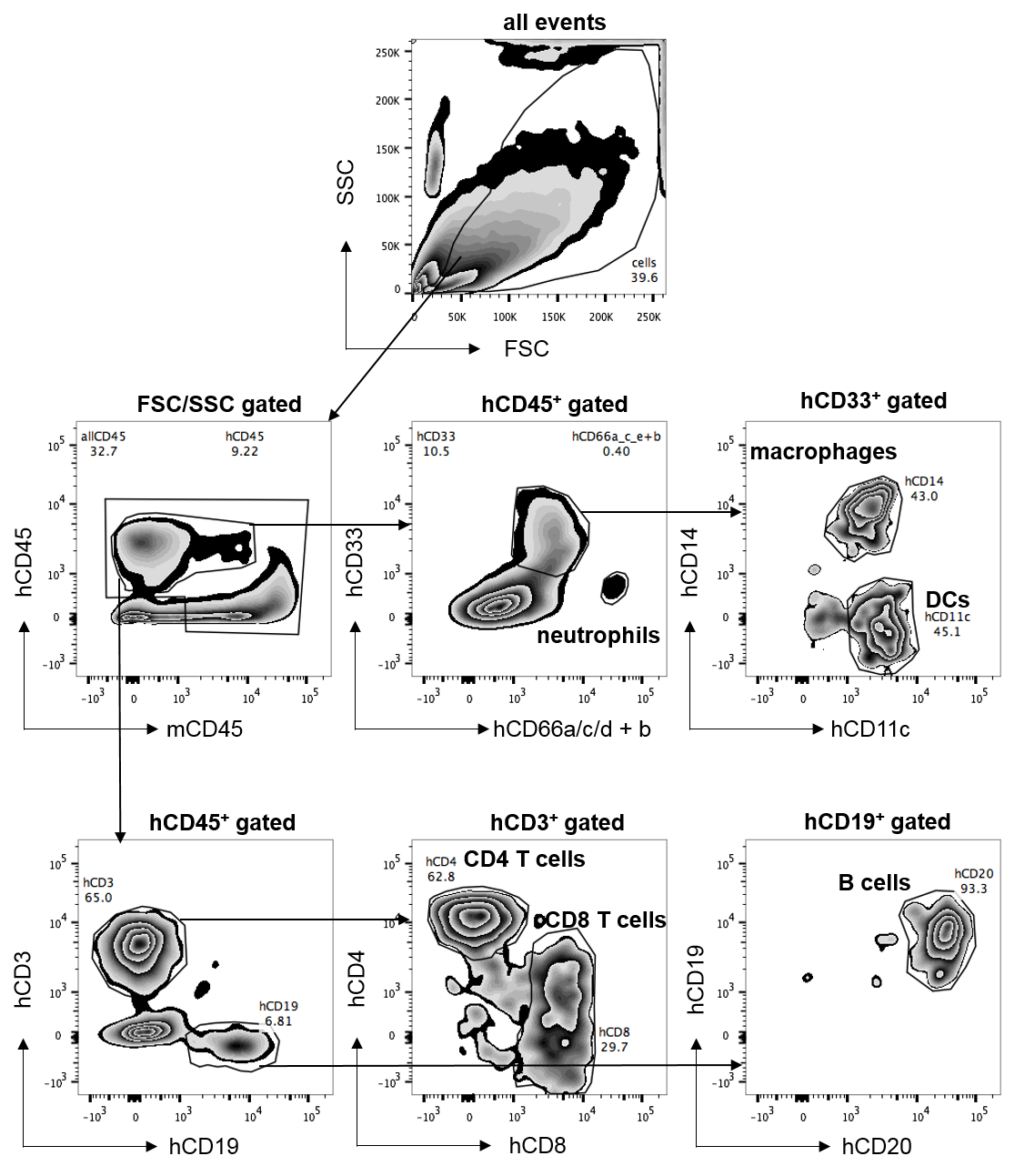
**

**Supplementary Figure 4. Flow cytometric gating strategy of human innate and adaptive immune cells in HIS-NSG lungs.** Representative dot plots showing flow cytometric gating strategy applied to identify human innate and adaptive immune cells (FSC/SSC leukocytes→ human CD45^+^ granulocytes → human CD33^+^CD66^+^a/c/e/b neutrophils; FSC/SSC leukocytes→ human CD45^+^ monocytes→ human CD33^+^CD14^+^ monocytes/macrophages; FSC/SSC leukocytes→ human CD45^+^ monocytes→ human CD33^+^CD11c^+^ dendritic cells; FSC/SSC leukocytes→ human CD45^+^ lymphocytes → human CD3^+^ T cells and CD19^+^ B cells; human CD3^+^ T cells→ CD4^+^ T cells and CD8^+^ T cells; human CD19^+^ B cells → human CD20^+^ B cells).

**
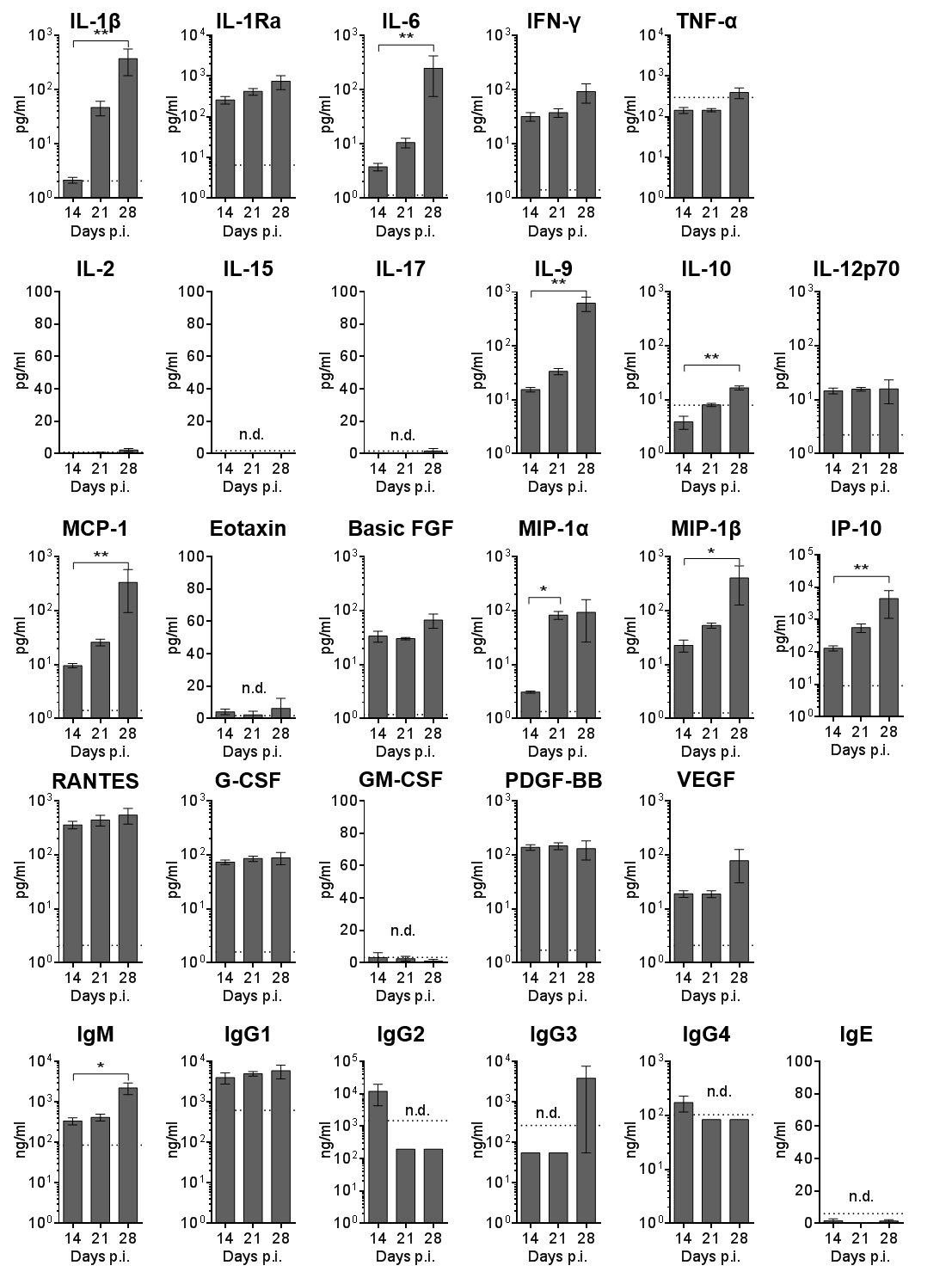
**

**Supplementary Figure 5. Human biomolecule expression in Mtb infected HIS-NSG lungs.** Human biomolecules were determined in lung homogenates of HIS-NSG mice. Bar graphs showing protein levels at 14, 21 and 28 days p.i. *n* = 4 - 5 mice per time point, Mean ± SEM, Kruskal-Wallis/Dunn’s multiple comparisons test. n.d., not detected/below detection limit, dotted line: detection limit; ** *P* < 0.01, * *P* < 0.05.


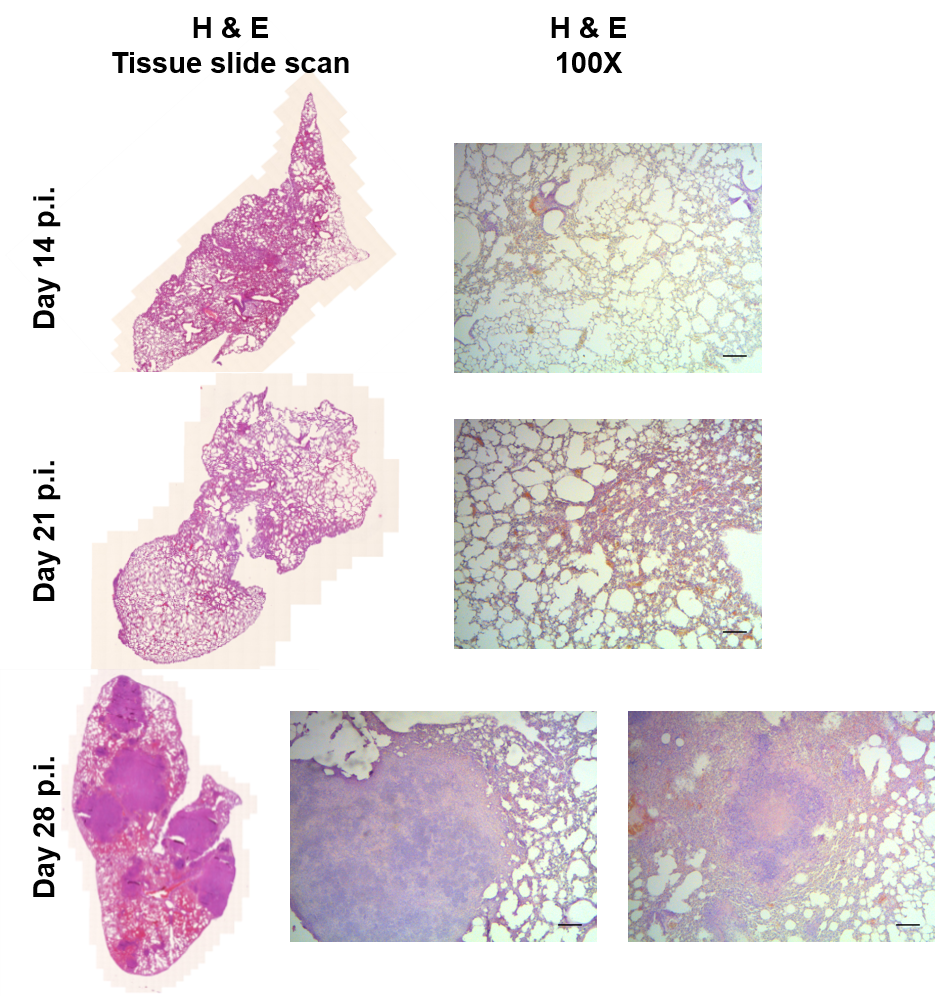


**Supplementary Figure 6. Changes in HIS-NSG lung architecture as TB disease progresses.** Entire left lung lobes collected from representative HIS-NSG mice at 14, 21 and 28 days post Mtb infection show increased loss of spongy architecture as TB progressed. Hematoxylin and Eosin stain of tissue slide scans; Hematoxylin and Eosin stain 100X, scale bar = 200 µm.
